# Impact of sodium alginate hydrogel containing bacteriophage peptides that specifically bind to the EtCab protein on the inhibition of *Eimeria tenella* infection

**DOI:** 10.1186/s13567-024-01425-4

**Published:** 2025-01-21

**Authors:** Hang Chen, Wenjing Zhi, Bingrong Bai, Faisal R. Anjum, Zhipeng Jia, Rui Kong, Qiuju Liu, Biao Wang, Chunli Ma, Dexing Ma

**Affiliations:** 1 College of Veterinary Medicine, Northeast Agricultural University, Harbin, China; 2 Bristol Veterinary School, University of Bristol, Langford, Bristol, UK; 3 College of Food Science, Northeast Agricultural University, Harbin, China; 4 Heilongjiang Provincial Key Laboratory of Pathogenic Mechanism for Animal Disease and Comparative Medicine, College of Veterinary Medicine, Northeast Agricultural University, Harbin, China

**Keywords:** *Eimeria*, *Et*Cab, phages, peptide, encapsulation, protection

## Abstract

Avian coccidiosis, caused by the protozoan *Eimeria,* leads to significant economic losses for the poultry industry. In this study, bacteriophages that specifically bind to the calcium-binding protein (*Et*Cab) of *Eimeria tenella* were selected using a biopanning process with a pIII phage display library. The recombinant *Et*Cab protein served as the ligand in this selection process. The binding ability of target phages to the *Et*Cab protein or *E. tenella* sporozoites was evaluated. The role of peptides corresponding to target phages in inhibiting the invasion of *E. tenella* sporozoites into cells was analysed using flow cytometry. Subsequently, the phages were encapsulated in sodium alginate to protect them from degradation in gastric fluid, which has a low pH value. Chickens were orally administered both microencapsulated and non-microencapsulated phages, and the protective effects against *E. tenella* infection were assessed. The binding mechanism of these peptides to the *Et*Cab protein was investigated through in silico analysis. The results indicated that three specific phages (Y, G, and V) could bind effectively to recombinant *Et*Cab protein as well as to sporozoite proteins. All three peptides, particularly Y and G, demonstrated significant inhibition of sporozoite invasion into cells in vitro. Additionally, oral administration of the encapsulated phages Y and G provided a higher level of protection against *Eimeria* infection compared to encapsulated phage V and the unencapsulated phages. Molecular docking studies revealed that three peptides, particularly Y and G, efficiently bind to the *Et*Cab protein through hydrogen bonds. This study provides a reference for developing small molecular drugs targeting coccidiosis.

**Supplementary Information:**

The online version contains supplementary material available at 10.1186/s13567-024-01425-4.

## Introduction

Avian coccidiosis is an intestinal disease in poultry caused by one or more species of the protozoan genus *Eimeria*. These *Eimeria* parasites invade the intestinal epithelial cells, leading to acute enteritis and tissue necrosis, which manifest as clinical symptoms of diarrhoea, dehydration, and weight loss [1, 2].

Among the seven known *Eimeria* species, *Eimeria tenella* predominantly targets cecal epithelial cells, resulting in cecal hemorrhagic lesions. The life cycle of *Eimeria* consists of two main stages: the exogenous phase (sporogenesis) and the endogenous phases (schizogenesis and gametogenesis) [3].

During sporogenesis in the intestinal tract, sporozoites are released from sporulated oocysts. These sporozoites attach to the intestinal epithelial cells, initiating a complex invasive process in which a variety of sporozoite-secreted proteins are involved [2]. In the schizoite phase, merozoites develop from sporozoites and progress through intestinal epithelial cells, developing into large and small gametocytes. This process is followed by sexual reproduction to form zygotes, which develop into oocysts [2]. These oocysts are released from the intestinal epithelial cells and excreted with the intestinal contents. Under suitable environmental conditions, the oocysts develop into sporulated oocysts. A new cycle of sporogenesis begins when chickens orally ingest these sporulated oocysts.

The long-term use of anticoccidial drugs has led to the emergence of drug-resistant parasites, posing a significant challenge to the global poultry industry [4]. Additionally, the presence of drug residues in poultry products raises potential concerns for food safety and human health [5]. Live attenuated vaccines also present challenges for poultry health, as large-scale infections may lead to outbreaks due to the reversion of virulence in attenuated vaccines.

These drawbacks associated with traditional control methods highlight the urgent need to develop novel, cost-effective, and safe preventive measures to protect the health and well-being of chickens worldwide.

*Eimeria* is a site-specific intracellular parasite that requires adhesion to the surface of host cells for subsequent invasion [6]. Various proteins have been identified as important for this invasion, including microneme (MIC) proteins like *E. tenella* MIC3 (*Et*MIC3) [7] and apical membrane antigen 1 (AMA1) [8, 9]. Recent studies have shown that calcium ions (Ca^2+^) are essential for intercellular communication, and calcium-binding proteins (CBPs) play a significant role in regulating various cellular functions [10].

CBPs exhibit several structural characteristics, including a specific Ca^2+^ binding structural domain known as EF-Hand, which features a helix-loop-helix structure and is highly conserved across different CBPs [11].

Research has shown that certain CBPs located on the surface of parasites play a significant role in the adhesion and invasion of protozoan parasites. For instance, *Toxoplasma gondii* CDPK1 (TgCDPK1) is involved in adhesion and invasion by regulating the secretion of TgMIC2 [12], while TgCDPK4 may modulate the invasion of these parasites [13]. Similarly, *Plasmodium falciparum* CDPK1 (PfCDPK1) is crucial for regulating merozoite motility during egress and invasion [14]. Recently, the *E. tenella* Cab (*Et*Cab) protein has been identified as essential for adherence during its invasion of host cells [15].

M13 phages were utilised to display various exogenous proteins or peptides on the surface of pIII capsid proteins; this is known as phage display technology [16]. The results showed that functional peptides screened from a phage display library, using sonicated sporozoites of *E. acervulina* and *E. tenella* as affinity ligands, can specifically bind to sporozoites. This binding effectively inhibits their invasion into host cells [17].

Additionally, a phage display library displaying single-chain variable fragments (scFv) was constructed to identify peptides with a strong ability to bind specifically to *E. tenella* sporozoites and merozoites [3, 18]. In the current study, we focus on the crucial role of the *Et*Cab protein in sporozoite invasion into host cells. We hypothesise that peptides that specifically bind to the *Et*Cab protein could effectively inhibit this invasion.

To test this hypothesis, four rounds of biopanning were conducted using twelve peptide libraries, with the *Et*Cab protein serving as a ligand. Furthermore, since it has been reported that biocompatible material such as sodium alginate can protect encapsulated biomaterials from degradation, we examined the effect of the sodium alginate hydrogel that encapsulates bacteriophage peptides specifically binding to the *Et*Cab protein. This effect was investigated against *E. tenella* sporozoite invasion both in vivo and in vitro. Additionally, a three-dimensional structural model of the *Et*Cab protein was constructed, and molecular docking between the affinity peptide and the *Et*Cab model was analysed.

## Materials and methods

### Animals and parasites

One-day-old specific pathogen-free (SPF) chickens were obtained from the Harbin Veterinary Research Institute in Heilongjiang, China, and raised in a coccidia-free environment. The *E. tenella* strain used in this study was maintained in our laboratory, and sporulated oocysts of *E. tenella* were passaged in chickens every three months. The collection of sporulated oocysts and the preparation of purified sporozoites were conducted as described previously [19].

All animal experiments were carried out at the Veterinary Hospital of Northeast Agricultural University in China. The experimental protocols adhered to the regulations set by the Ethics Committee for Animal Sciences at Northeast Agricultural University, Heilongjiang Province, China (NEAUEC20220335).

### Preparation of rabbit antisera against *Et*Cab

The rabbit antisera against the *Et*Cab protein was prepared with some modifications based on the previously reported methods [7]. The plasmid pUC57-*Et*Cab (preserved in our laboratory; the GenBank accession number for *Et*Cab is XP_013229050.1) was digested using the restriction enzymes *Bam*H I and *Xho*I. The resulting *Et*Cab gene fragment was ligated into a *Bam*H I/*Xho*I-digested pET-30a(+) vector. The positive plasmid pET-30a(+)-*Et*Cab was then transformed into *E. coli* BL21 (DE3).

The expression of the *Et*Cab protein in the supernatant from the ultrasonic lysate of recombinant positive bacteria was analysed using sodium dodecyl sulfate–polyacrylamide gel electrophoresis (SDS-PAGE). The purification of *Et*Cab protein was conducted using nickel (Ni^2+^)-nitrilotriacetic (NTA) His Bind Resin (Merck, Darmstadt, Germany) following the established methods [8]. SDS-PAGE subsequently verified the purification of the target protein.

An emulsion was prepared by mixing 1.0 mg of purified *Et*Cab protein with an equal volume of Freund’s complete adjuvant (Sigma Aldrich, StLouisMO, USA), serving as the immunogen for immunising New Zealand white rabbits (provided by the Harbin Veterinary Research Institute, Harbin, China) through subcutaneous injection. One week later, the rabbits were given another dose of recombinant *Et*Cab protein (1.0 mg) and an equal volume of Freund’s incomplete adjuvant (Sigma Aldrich, USA). The following two immunisations were conducted at weekly intervals.

After the final immunisation, serum was collected and stored at −20 °C. The titer of rabbit polyclonal antisera against the *Et*Cab protein was measured using an indirect enzyme-linked immunosorbent assay (ELISA), as described in a previous report [20].

### Specific detection of antisera for the *Et*Cab protein

Western blot was used to evaluate the specificity of rabbit antisera against the natural *Et*Cab protein in *E. tenella* sporozoites. The methods for purifying sporozoites, as well as sonication and western blotting, were performed as previously described [21]. Additionally, an indirect immunofluorescence assay (IFA) was employed to demonstrate that the prepared antisera specifically reacted with the *Et*Cab protein on the surface of *E. tenella* sporozoites.

In brief, 1.0 × 10^5^ MDBK cells were seeded into each well of a 96-well plate. Once the cells reached 50% confluence, 3.0 × 10^5^ sporozoites were added to each well and incubated for 4 h. Following incubation, the wells were washed three times to remove any uninvaded sporozoites. Next, 1.0 mL of 4% paraformaldehyde was added to fix the cells for 40 min. After another washing step, 1 mL of glycine (0.1M/L) was added to each well and incubated for 15 min. Then, 1 mL of Triton X-100 (0.1%) was added to each well and incubated at 37 ℃ for 15 min.

The cells were then incubated with rabbit anti-*Et*Cab protein polyclonal sera (1:100) at 4 ℃ overnight. Following this, the cells were treated with fluorescein isothiocyanate (FITC)-conjugated goat anti-rabbit IgG (1:100) (Solarbio, Beijing, China) for 2 h. DAPI was added and allowed to incubate for 20 min. Fluorescence images were captured using the EVOS M700 inverted fluorescence microscope (Thermo, Massachusetts, USA).

### Screening for phages that specifically bind to *Et*Cab

The process for screening *Et*Cab protein affinity phages was conducted as outlined in previous reports and followed the protocols provided in the phage display peptide library kit from New England Biolabs, USA. The size of the phage library was 1.0 × 10^9^, and each phage displayed five identical peptides on its surface.

In brief, 100 μL *Et*Cab protein (at a concentration of 100 μg/mL) was coated in each well of a 96-well plate. After incubating overnight at 4 ℃, the plate was blocked with 2% BSA for 2 h and then washed five times with Tris buffer containing 0.1% Tween-20 (TBST, pH 7.4). Next, 100 μL of the phage display library (at a 1:100 dilution, totalling 1.5 × 10^14^ pfu) was added to the wells and incubated at 25 ℃ for 30 min. The plate was then washed five times with TBST and once with 0.2 mol/L glycine–HCL (pH 2.2). After a 10-min incubation at 25 ℃, 1.0 mol/L Tris–HCL (pH 9.1) was added to neutralise the PH.

The eluent collected from the 96-well plate constituted the first round of biopanning bacteriophages. The titer of the target phage was determined and amplified in *E. coli* ER2738 according to the instructions provided in the phage display peptide library kit (New England Biolabs, USA). The subsequent three rounds of biopanning were conducted using the same method, with the concentration of Tween-20 in TBST increased to 0.5% and the number of washes increased to 20 times.

In the fourth round, the amplified bacteriophages were again cultured in *E. coli* ER2738, and after titration, 30 randomly selected bacteriophages were stored at −20 °C for more analysis.

### Titre determination of target phages

Bacteriophage titre was determined using a modified double agar overlay plaque assay [22]. In brief, LB medium was utilised to dilute the phages to a suitable concentration, ranging from 10^8^ to 10^11^ for amplified-PEG/NaCl concentrated phage culture supernatants and from 10^1^ to 10^4^ for unamplified panning eluates.

To start the assay, 10 μL of the phage was added to 200 μL of the host strain ER2738 (OD_600_ = 0.5) during its logarithmic phase, and the mixture was incubated with shaking at room temperature for 10 min. The infected bacteria were added to 3 mL of top agar medium, mixed immediately, and poured over preheated LB/IPTG/Xgal medium at 37 °C. After allowing the mixture to cool for 10 min, the plate was inverted and cultured at 37 °C overnight.

$${\text{The formula for calculating titer is}}:{\text{ pfu}}/{\text{mL}}\, = \,{\text{Number of plaques}}\, \times \,{1}00\, \times \,{\text{reciprocal of counted dilution}}.$$

The formula for calculating titer is: pfu/mL = Number of plaques × 100 × reciprocal of counted dilution.

### The ability of selected phages to bind to the *Et*Cab protein

The binding capability of selected phages to the *Et*Cab protein was assessed using ELISA, following previously established protocols [7]. The plate was coated with 10 μg of *Et*Cab protein per well and incubated overnight. After incubation, the plate was washed with PBST containing 0.5% Tween-20 and then blocked with 5% skim milk. Following this, 1.0 × 10^12^ pfu of the selected phages were added and incubated for 2 h.

The primary antibody, rabbit anti-M13 polyclonal antibody, was then used, diluted to 1:3000. For detection, a secondary antibody, goat anti-rabbit IgG (1:1000) conjugated with horseradish peroxidase (HRP) from Sigma (USA), was applied. Subsequently, a substrate solution containing 0.01% H_2_O_2_ and 1 mg/mL o-phenylenediamine was subsequently added for the reaction, which was then terminated using 2 M sulfuric acid. The OD_450_ value for each well was recorded.

For control measures, phages that bound to the N protein of infectious bronchitis virus (IBV), stored in our lab, were used as a negative control, while phages binding to the *Et*MIC3 protein were designed as the positive control [7]. Each phage detection assay was performed in triplicate.

### Sequence analysis of target phages

DNA from selected high-affinity phages was extracted, and PCR amplification was conducted using the primer pair provided in the phage display peptide library kit from New England Biolabs, USA. After observing the target bands, the PCR product was sequenced, determining twelve peptide sequences corresponding to the target phages. The affinity peptides were then synthesised by GenScript Co., Ltd (Nanjing, China).

### Binding of *Et*Cab protein to target phages

To evaluate the binding capability of the recombinant *Et*Cab protein and sporozoite protein to target phages, both ELISA and competitive ELISA were conducted as previously described [7].

To determine the binding capability between *Et*Cab protein and target phages, 100 μL of affinity phages, serially diluted from 1 × 10^12^ to 1 × 10^6^ pfu, was added to a 96-well plate and incubated overnight. After blocking, each well received 100 μL of purified *Et*Cab protein at a 100 μg/mL concentration, followed by 100 μL of HRP-labeled goat anti-rabbit IgG. The OD_450_ value was measured in triplicate for each sample.

For the competitive ELISA, 50 μL of rabbit anti-*Et*Cab antiserum was diluted at concentrations of 1:1000, 1:1500, 1:2000, and 1:2500, corresponding to 60, 40, 30, 24, and 20 μg/mL, respectively. This was combined with 50 μL of *Et*Cab protein (100 μg/mL) as the binding competitor and added to each well containing 100 μL of coated positive phage (1.0 × 10^12^ pfu). After incubation, OD_450_ values were recorded, and each sample was tested in triplicate. The negative and positive phages described above were included as controls.

The binding capability of sporozoites protein to the target phages was assessed using the same method as described earlier. However, in this process, 100 μL of sonicated (300 W, 5s, 5s intervals) sporozoites (1.0 × 10^6^/mL) protein (1.2 mg/mL) was added to interact with the coated target phages. Each sample was tested in triplicate. The negative and positive phages mentioned previously were included as controls.

### Purification and labelling of sporozoites

The purification of *E. tenell*a sporozoites was carried out using the previously reported method [7]. First, preserved sporulated *E. tenella* oocysts were washed and treated with a sodium hypochlorite solution. The targeted oocysts were then collected, resuspended, washed and agitated with glass beads to break the oocyst wall. The released sporocysts were collected, washed and resuspended before treatment with 0.25% trypsin and 4% taurocholic acid sodium salt to complete the excystation process. The purified sporozoites were obtained by centrifuging the samples in Percoll (55%).

Next, 5.0 × 10^6^ sporozoites were labelled with CFDA-SE (Beyotime, China) according to the provided protocols. The labelled sporozoites were then cultured in DMEM medium (Gibco, Leicestershire, UK) at 37 °C for 30, 60, and 90 min. Labelling efficiency was assessed using a fluorescence microscope (Nikon) and flow cytometry.

### Inhibition of sporozoites invasion into cells invitro

To determine the maximum non-toxic concentration of the three synthesised peptides on MDBK cells, the cells were incubated with the peptides at concentrations of 125, 250, 500, and 1000 μg/mL for 2 h at 37 °C in a humidified atmosphere containing 5% CO_2_. After each incubation period, the medium was discarded, and the cells were washed three times with PBS. Subsequently, serum-free DMEM (100 μL/well) and CCK-8 reagent (10 μL/well) were added for 1 h at 37 °C. The absorbance of each well was measured using an ELISA reader (BioTek, Elx808), and cell viability was calculated. Additionally, any morphological changes in the cells were observed.

The purification and labelling of sporozoites with CFDA-SE were performed using the abovementioned method. DMSO was utilised to dilute the three affinity peptides to concentrations of 125, 75, and 25 μg/mL and to dilute rabbit anti-*Et*Cab polyclonal serum at ratios of 1:100, 1:200, and 1:300. The sporozoites were incubated with either the polyclonal serum or the affinity peptides in a cell culture plate for 1 h at 37 °C and 5% CO_2_, after which they were collected by centrifugation. The harvested sporozoites were then added to a monolayer of MDBK cells (1.0 × 10^5^ per well). Following 4 h of incubation, the cells were washed three times with PBS to remove uninvaded sporozoites and then digested with trypsin (0.25%). The green fluorescence signal in the intracellular MDBK cells was detected by flow cytometry to calculate the sporozoite invasion rate. The invasion rate and inhibition rate were calculated using the following formulas:

$${\text{Invasion rate}}\, = \,\left( {\text{count of cells with green fluorescence}} \right)/({\text{count of cells with and without fluorescence}}).$$

Invasion inhibited rate = [1−(count of cells in the group treated with target peptides or anti-*Et*Cab antisera**/**count of cells in the group without treatment with peptides or anti-*Et*Cab antisera)] × 100%.

### Release characteristics of target phages from microspheres in both in vivo and in vitro conditions

To prepare phage-encapsulated microspheres, 100 μL of affinity phage (1.0 × 10^13^ pfu) was mixed thoroughly with 10 mL of a 1.5% sodium alginate solution. This mixture was then added dropwise to a 0.1 mol/L calcium chloride solution using a syringe while stirring continuously for 1 h to allow the microspheres to harden. The encapsulation efficiency (EE) was calculated using the formula: EE = (entrapped phages/total phages) × 100%.

To test the stability of the microspheres, 100 mg of microspheres and 100 μL of free phages were combined with 900 μL of simulated gastric fluid (SGF), which consisted of 154 mM NaCl and 0.3% (w/v) pepsin at pH2.0 [23]. The mixture was gently agitated at 41 °C. Samples of the microspheres were taken at 10, 30, 60, 90, and 120 min, washed with SM buffer (0.25 g MgSO_4_·7H_2_O pH 7.5) [24], and dissolved in 1.0 mL of a microcapsule-broken solution (MBS) containing 50 mM sodium citrate tribasic dehydrate and 200 mM sodium bicarbonate in a 50 mM Tris–HCl buffer at pH 7.5 [24]. The phage titers were then measured.

The release of target phages from the microspheres in simulated intestinal fluid (SIF), which contained 0.3% (w/v) bile salt, 111 mM NaCl, 11 mM KCl, 2 mM CaCl_2_, 16 mM NaHCO_3,_ and 1% (w/v) trypsin at pH 7.0 [23], was assessed as follows. 100 mg of microspheres were incubated in SGF (pH 2.0) at 41 °C for 2 h. After washing with SM buffer (pH 7.5), the microspheres were transferred to a new tube containing 900 μL SIF (pH 6.7) and incubated at 41 ℃. At intervals of 0, 5, 15, 30, 60, 90, and 120 min, 100 μL samples were taken to measure phage titers. The incubation was terminated by adding SM buffer (pH 7.5), and a phage release curve was plotted based on the phage titer and time. Each sample was tested in triplicate.

To measure the distribution of target affinity phages in the intestinal tract of chickens, sodium alginate microspheres encapsulating the target phages and free phages were orally administered to the chickens at a dose of 1.0 × 10^10^ pfu. At 1 and 2 h post-oral administration, contents from the gastric, duodenum, ileum, jejunum and cecal regions were collected and placed in MBS solution (pH 7.5). The phage titers were measured and curves depicting phage titers at different intestinal sites at different times were drawn. The phage titers were calculated using the formula: titers of phages = log_10_ (number of phage plaques × dilution ratio × total volume of sample ÷ 10). The value 10 indicates that the number of phage plaques is derived from a 10 µL diluted sample when only one phage plaque is observed on a plate made with stock solution without any dilution. The formula is as follows: log_10_ (1 × 1 × 2500/10) = 2.39794, representing the lower detection limit.

### In vivo anticoccidial effects of target phages

At 10 days of age, the chickens were randomly divided into nine groups, with 10 chickens in each group. At 21 days of age, all groups, except for group 1 (TBS group), were orally challenged with 1.0 × 10^4^ *E. tenella* sporulated oocysts. On days 0, 1, and 2 post-challenge, the chickens in group 2 (phage Y + Alg), 3 (phage G + Alg), and 4 (phage V + Alg) were orally gavaged with 1.0 × 10^10^ pfu of phage Y, G, and V encapsulated in sodium alginate microspheres, respectively. Chickens in groups 5 (phage Y), 6 (phage G), and 7 (phage V) received 1 × 10^10^ pfu of free phage Y, G, and V without encapsulation. Group 8 received sodium alginate microspheres (Alg), while group 9, the *E. tenella* challenge control, was given TBS (pH7.2).

The chickens in each group were weighed both before the *E. tenella* challenge and on day 7 post-challenge to calculate body weight gain (BWG). Faecal samples were collected from individual chickens in each group between days 5 and 7 post-challenge to calculate oocysts per gram (OPG). Additionally, cecal lesion scores and oocyst reduction rates were recorded as previously described [19].

All animal experiments followed the regulations set by the Ethics Committee for Animal Sciences at Northeast Agricultural University, Heilongjiang Province, China (NEAUEC20220335).

### Histopathological examination in cecal tissues

Cecal tissues from chickens in each group were collected, rinsed gently with PBS, and stored in 10% formalin for histopathological examination. After 1 week of fixation, the tissue specimens were trimmed, rinsed with tap water overnight to remove the fixative, and then dehydrated using alcohol of different concentrations. Subsequently, the tissues were clarified in xylene and embedded in paraffin. The paraffin-embedded block was cut into 4 μm thick sections, stained with hematoxylin and eosin (H&E staining) [25], and finally mounted with neutral resin for observation and photography.

### Homologous modelling, molecular docking, and homology analysis

The protein-coding gene sequence for EtCab was obtained from the UniProt database. Protein secondary structure analysis was conducted using the online SOPMA software [26]. The 3D structure of the target sequence was predicted using the I-Tasser server, which recognised the template sequence through Lomets [27]. This server was also applied to optimise the models [28]. Online software was utilised to evaluate the stereochemical characteristics of the constructed models [29].

Homologous modelling of the three target peptides was performed using Modeller 10.1 software, further optimised through the Chiron portal. The ClusPro [30] website was used for the flexible docking of proteins and target peptides, while pymol was employed for drawing and analysis. Sequence comparison was conducted using ESPript3.0 [31].

Sequences used for analysing membrane-associated calcium-binding proteins from *E. necatrix* (CDJ63198.1), *E. acervulina* (XP_013252173.1), *E. mitis* (XP_013357999.1), and *E. maxima* (XP_013337521.1) were retrieved from GenBank.

### Statistics and analysis

Statistical analysis was conducted using SPSS software v26.0 (SPSS Inc., Chicago, IL, USA). The analysis involved a one-way analysis of variance (ANOVA) along with Duncan’s multiple-comparison procedures. Data were presented as mean ± SD. A *p*-value of less than 0.05 was considered statistically significant, while a *p*-value of less than 0.01 was deemed highly significant.

## Results

### Characterisation of polyclonal antisera against *Et*Cab protein

**Figure 1:**
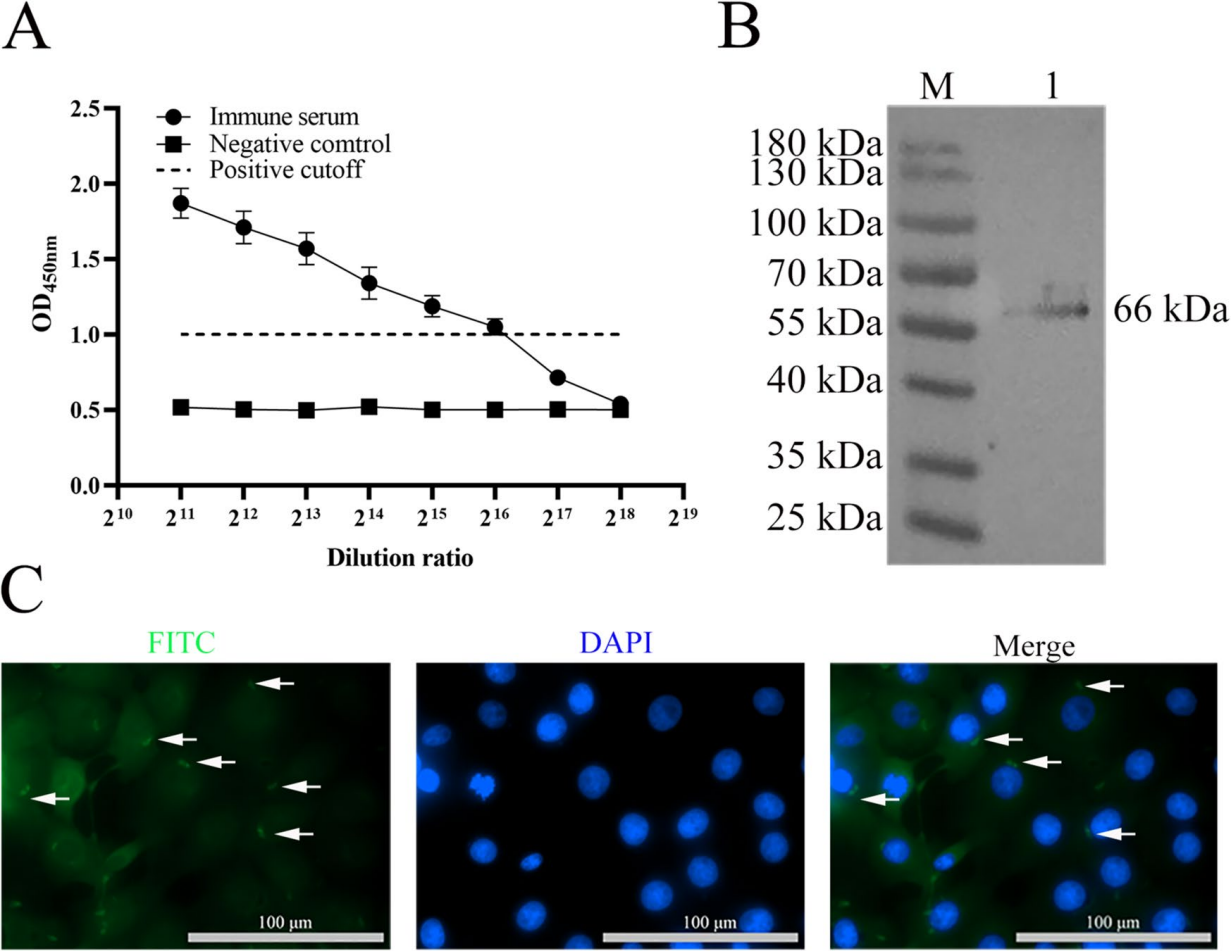
**Detection of prepare**d **polyclonal antisera against EtCab protein.** The titre of the prepared antisera against the EtCab protein was 1:2^16^. The positive cutoff = 2 × OD_450_ value of negative control (**A**). Western blot analysis was performed to detect the specific reaction of the anti-EtCab antisera with *E. tenella* sporozolites, revealing a band of 66 kDa (**B**). An indirect immunofluorescence assay (IFA) showed that the anti-EtCab antisera specifically reacted with the EtCab protein of *E. tenella* sporozoites, producing a green fluorescence signal (**C**). The titre of each sample was tested in triplicate, and the values are presented as mean ± SD (*n* = 5).

The titre of the prepared polyclonal antisera against the *Et*Cab protein was 1: 2^16^ (Figure 1A). Western blot analysis demonstrated that the antiserum specifically reacted with *E. tenella* sporozoites, producing a single protein band at 66 kDa (Figure 1B). Indirect immunofluorescence assays indicated that the antisera against the *Et*Cab protein effectively reacted with *E. tenella* sporozoites as they invaded MDBK cells, confirmed by a distinct green fluorescence signal (Figure 1C).

### Screening of phages that bind to *Et*Cab and conducting binding assays

**Figure 2:**
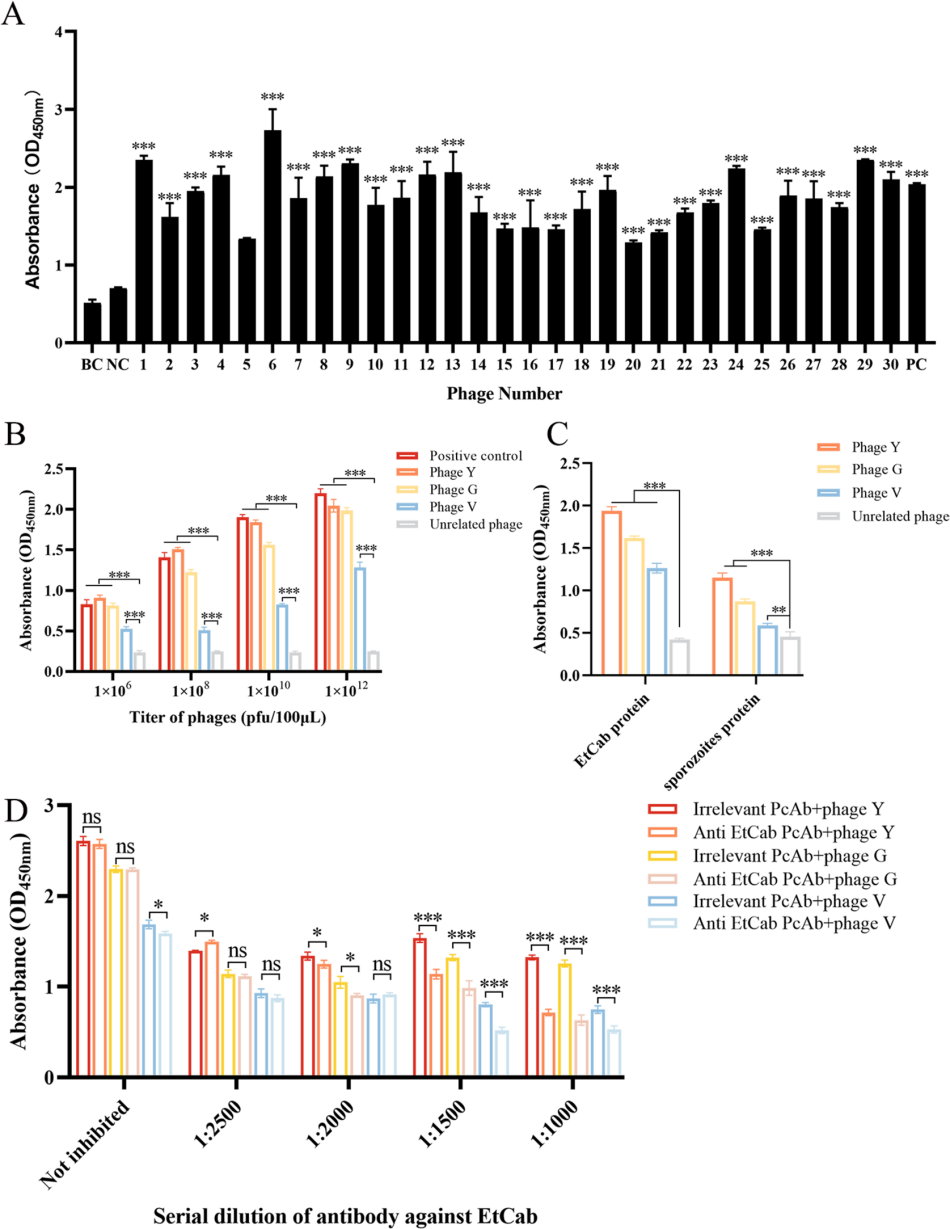
**Affinity determination of screening bacteriophage with EtCab protein.** The binding capability of thirty randomly selected phages was found to be higher than that of the blank control (BC) and the negative control (NC). The positive control (PC) phages, designed to bind to the EtMIC3 protein, were prepared as shown in (**A**). Phages were diluted from 1.0 × 10^12^ to 1.0 × 10^6^ pfu/10 μL and coated in each well of a 96-well plate. The binding abilities of phages Y, G, and V were greater than that of the NC phages, with phages Y and G exhibiting higher binding than phage V (**B**). When 1.0 × 10^12^ pfu/10 μL of phages Y, G, and V were coated per well in a 96-well plate, their binding abilities to the recombinant EtCab protein and sporozoites lysates supposed those of the NC phages (**C**). The binding of the three phages to the EtCab protein was significantly inhibited by anti-EtCab antisera when compared to an irrelevant polyclonal antibody (PcAb) against the ΔHexon protein of fowl adenovirus serotype 4 (FAdV-4), which served as the negative antibody control (**D**). Each sample was tested in triplicate and the values represent the mean ± SD (*n* = 5). Statistical significance is denoted as follows: *P < 0.05, **P < 0.01, ***P < 0.001, and “ns” indicates no statistical difference.

The plaque-forming units per millilitre (pfu/mL) of the positive phages binding to the *Et*Cab protein were identified following four rounds of affinity screening (Table 1), indicating that the enriched phages could bind to the *Et*Cab protein. The results from the ELISA demonstrated that the binding ability of all thirty selected bacteriophages to the *Et*Cab protein was significantly higher than that of the blank control (BC) and the negative bacteriophage control (NC) (*P* < 0.001) (Figure 2A).

**Table 1:** **Titre determination of phage elution (pfu)**

	The first round	The second round	The third round	The fourth round
Input phage (pfu/mL)	1.5 × 10^13^	1.5 × 10^13^	1.5 × 10^13^	1.5 × 10^13^
Output phage (pfu/mL)	4.9 × 10^6^	4.8 × 10^6^	8 × 10^6^	2.1 × 10^6^
Amplified phage (pfu/mL)	3.7 × 10^12^	5.6 × 10^12^	2.8 × 10^13^	3.5 × 10^13^

The sequences of twelve peptides from the thirty corresponding bacteriophages are summarised in Table 2. Among these, three sequences—YLNPINHRLTNP (Y), GDRMNVIWDTFA (G) and VSNGFHLHLELR (V)—exhibited the highest repetition ratios and were selected for subsequent experiments.

**Table 2:** **The deduced amino acid sequences of the selected thirty positive phages clones**

Product number of binding phages clones to EtCab protein	Amino acid sequence of twelve peptides corresponding to affinity phage
1 #,6 #,7 #,10 #,13 #,14 #,15 #,17 #,20 #,22 #,24 #,27 #,28 #	YLNPINHRLTNP (Y)
2 #,3 #,5 #,8 #,11 #,12 #,18 #,19 #,21 #,25 #,26 #,29 #	GDRMNVIWDTFA (G)
4 #,9 #,16 #	VSNGFHLHLELR (V)
23 #	DKVVRSTIDHLN
30 #	GFNLQVRGDMAT

All three target phages Y, G, and V were able to bind to the *Et*Cab protein. However, the affinity of target phages Y and G for the *Et*Cab protein was significantly greater than that of phage V (*P* < 0.001) (Figure 2B). The binding ability of bacteriophages Y, G, and V to recombinant *Et*Cab protein and sporozoite ultrasonic lysates was significantly higher than that of unrelated bacteriophages (selected from the N protein of the infectious bronchitis virus) (*P* < 0.001) (Figure 2C).

Competitive ELISA results demonstrated that as the concentration of *Et*Cab antisera increased, the antibodies competed with the three affinity bacteriophages to bind to the *Et*Cab protein. In contrast, the unrelated rabbit polyclonal antibody against avian adenovirus serotype 4 (FADV-4) Hexon protein did not show dose-dependent inhibition (Figure 2D).

### Inhibition of sporozoites invasion into cells in vitro

**Figure 3:**
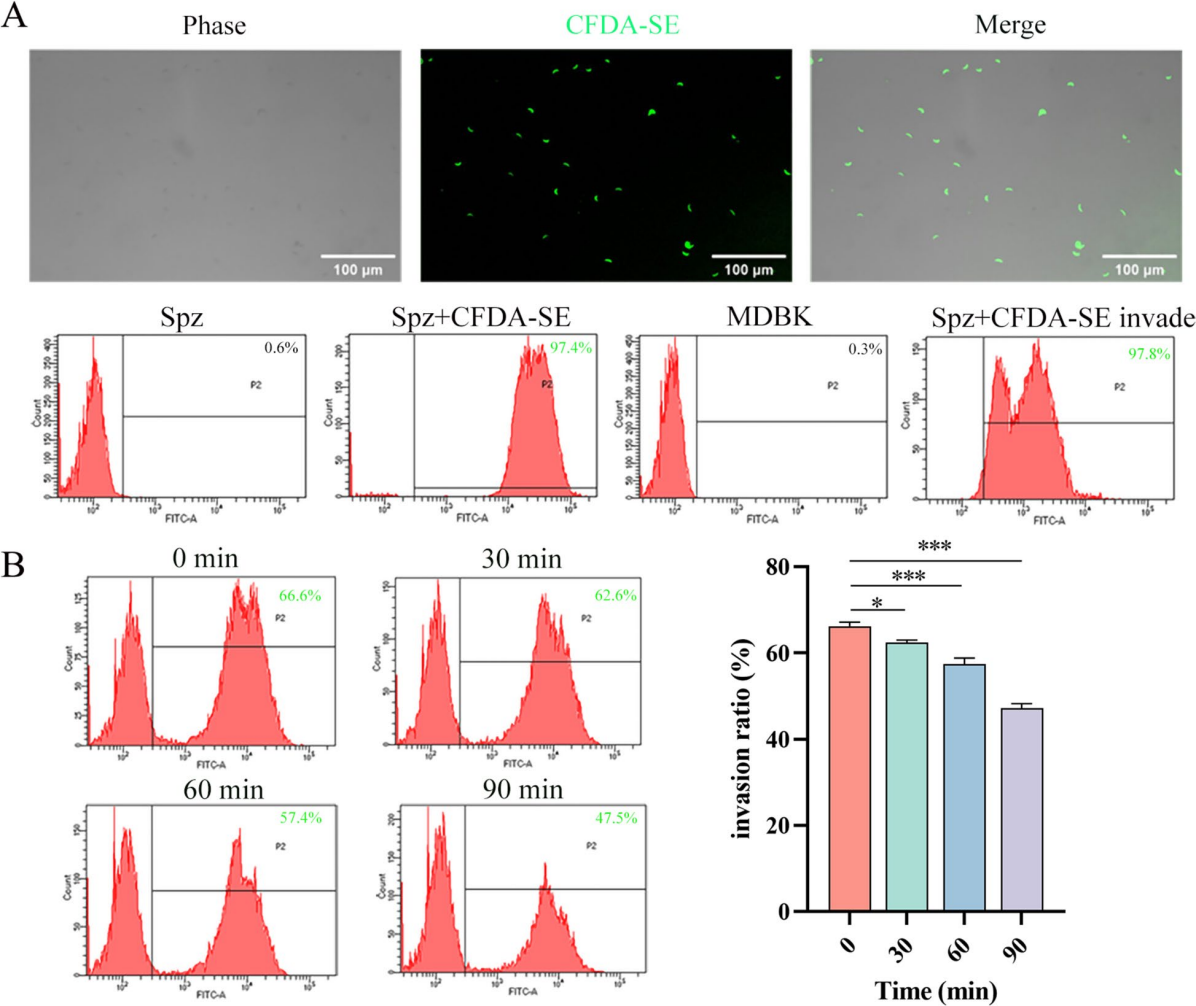
**In vitro model of *E. tenella* sporozoites invasion into MDBK cells.** Approximately 97.4% of freshly purified *E. tenella* sporozoites were labelled using CFDA-SE **(A)**. The labelled sporozoites were incubated in DMEM medium at 37 °C for time periods of 30, 60, and 90 min, before being co-incubated with MDBK cells for 2 h. Flow cytometry results indicated that as the duration of in vitro incubation of the sporozoites increased, the efficiency of their invasion into the cells decreased (**B**). Each sample was tested in triplicate, and the values are presented as mean ± SD (*n* = 3). Statistical significance is indicated as follows: *P < 0.05, **P < 0.01, ***P < 0.001, and “ns” indicates no statistical difference.

Fluorescence microscopy revealed that 97.4% of sporozoites were labelled with a fluorescent signal using the CFDA-SE kit. These labelled sporozoites could be detected in MDBK cells just 2 h after incubation, indicating their ability to invade MDBK cells (Figure 3A). However, the longer the sporozoites were incubated in vitro, the lower their invasion rate into MDBK cells became (Figure 3B).

**Figure 4:**
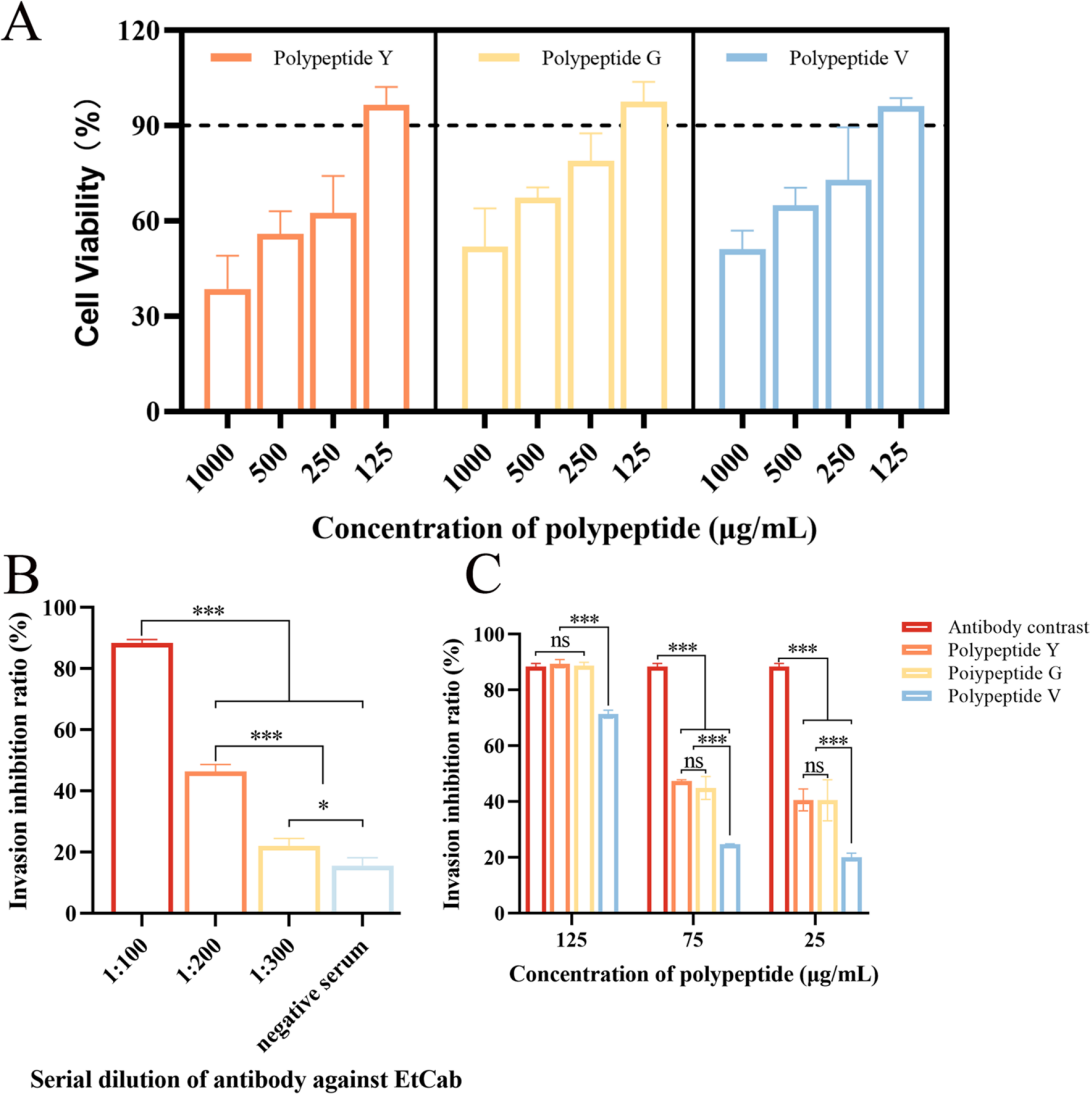
**Inhibition of sporozoites invasion into MDBK cells by target peptides in vitro.** The maximum non-toxic concentration of the three peptides was found to be 125 µg/mL (**A**). The labelled sporozoites were incubated with anti-EtCab antisera diluted 1:100, 1:200, and 1:300, respectively, before being added to MDBK cells. The dilution of anti-EtCab antisera at 1:100 resulted in the lowest invasion rate and the highest rate of invasion inhibition (**B**). Additionally, the labelled sporozoites were incubated with concentrations of 125, 75, and 25 μg/mL of the three peptides, and then introduced to MDBK cells. At a concentration of 125 μg/mL, peptides Y and G exhibited a higher rate of invasion inhibition (**C**). Each sample was tested in triplicate, and the values are expressed as mean ± SD (*n* = 3). Statistical significance is indicated as follows: *P < 0.05, **P < 0.01, ***P < 0.001, and “ns” indicates no statistical difference.

The maximum non-toxic concentration of the synthesised affinity peptides Y, G, and V for MDBK cells was 125 μg/mL (Figure 4A). Compared to negative sera, rabbit anti-*Et*Cab polyclonal antisera at various dilution ratios inhibited the invasion of *E. tenella* sporozoites into MDBK cells. Notably, the inhibitory effects diminished as the dilution ratio increased (Figure 4B).

All three affinity peptides demonstrated a significant ability to inhibit sporozoite invasion into MDBK cells in a dose-dependent manner. At every concentration tested, the inhibitory effects of peptides Y and G were significantly greater than that of V peptide (*P* < 0.001) (Figure 4C).

### Release and distribution of encapsulated target phages

**Figure 5:**
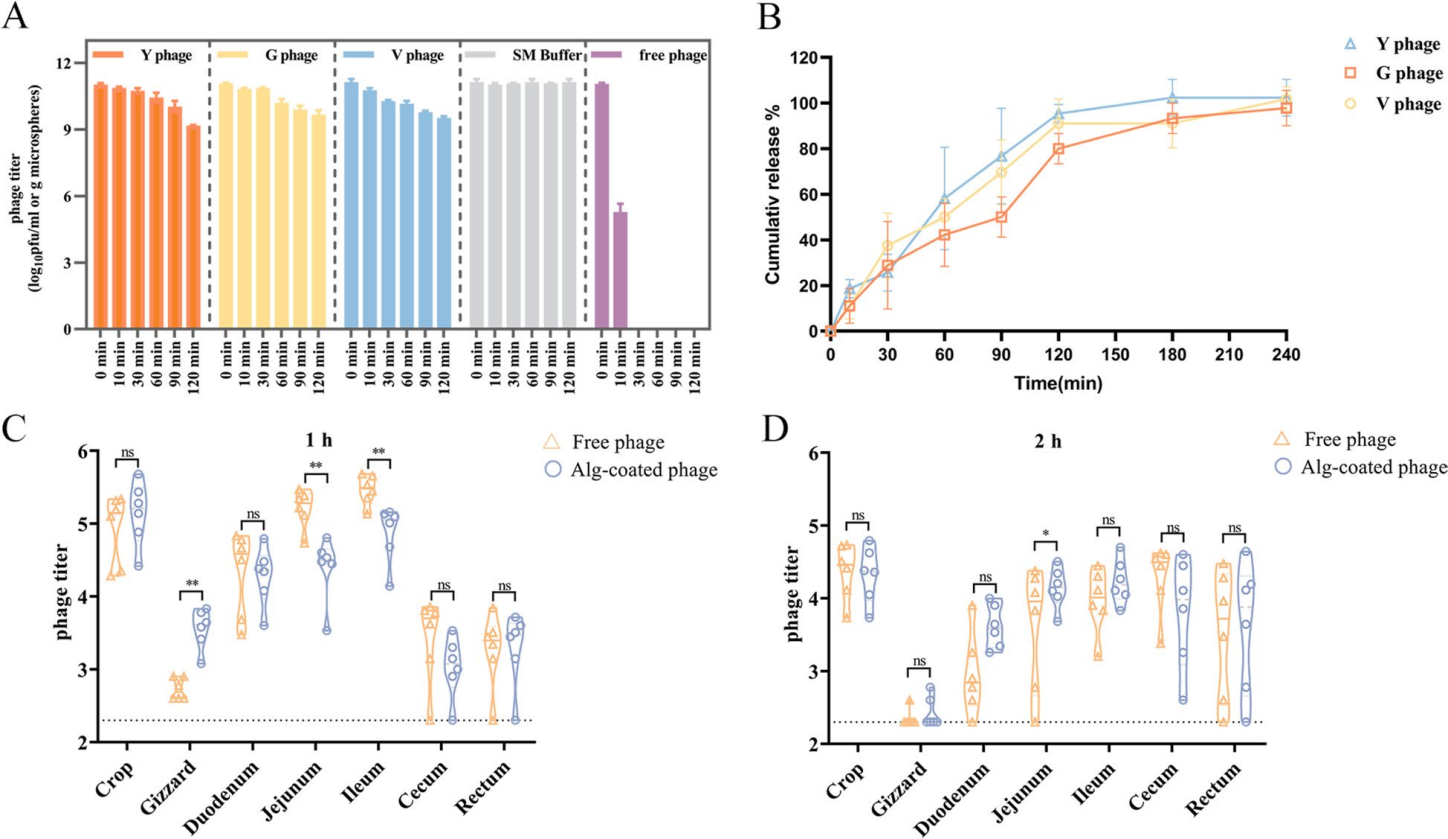
**Release and distribution of target phages encapsulated by sodium alginate.** Phages, both encapsulated and with sodium alginate (Alg-coated) and free (without encapsulation), were incubated in simulated gastric fluid (SGF) at pH 2. The free phages completely lost their activity after 30 min of incubation, while the titres of encapsulated phages decreased by only 1.62log_10_ pfu/g after 120 min (**A**). After being incubated in SGF for 2 h, the encapsulated phages transferred to simulated intestinal fluid (SIF) at pH 6.7 for further incubation. During this time, the titres of phages in the supernatant gradually increased. At 30 min, 32.9% of the encapsulated phages had been released, and at 120 min, 92.6% were released (**B**). 1 h after oral administration, the titres of phages in the duodenum, jejunum, and ileum were higher than those in the gizzard, cecum, and rectum. However, at 2 h post administration, the titres in the cecum were slightly higher than in the duodenum and gizzard (**C** and **D**). Values are expressed as mean ± SD (*n*** = **6). Statistical significance is indicated as follows: *P < 0.05, **P < 0.01, and “ns” indicates no statistical difference.

The encapsulation efficiency was 97.47 ± 4.62%. Free phages that were not encapsulated completely lost their activity after incubating in simulated gastric juice (pH 2.0) for 30 min. In contrast, the titre of phages coated with sodium alginate (Alg-coated) only decreased by 1.62 ± 0.24 log_10_ pfu/g after 120 min of incubation (Figure 5A). This indicates that the phages encapsulated in sodium alginate microspheres were protected from degradation in simulated gastric juice (pH 2.0).

When the Alg-coated microspheres were transferred from simulated gastric juice (pH 2.0) to simulated intestinal fluid (pH6.8), the titre of phages in the supernatant gradually increased. At 30 min, 32.9% of the encapsulated phages were released, and at 120 min, 92.6% were released (Figure 5B).

Following oral administration, the distribution of the target phage Y in the chicken digestive tract was evaluated, as shown in Figure 5C and D. The titre of target phages in the gizzard for the Alg-coated group was significantly higher than that of the free phage group at 1 h (*P* < 0.01), indicating that some unencapsulated phages were degraded after oral administration. Although the titres of free phages in the jejunum and ileum were significantly higher than those in the Alg-coated group (*P* < 0.01) at 1 h, the titre of phages in the jejunum in Alg-coated group at 2 h was significantly higher than that in the free phage group (*P* < 0.05). This suggests that phages encapsulated by sodium alginate could be gradually released and remain at high titres in the anterior digestive tract for a relatively long time.

### Evaluation of anti-coccidial effects in vivo

As shown in Table 3, the average body weight gain (WG) in the groups that received oral administration of encapsulated and unencapsulated bacteriophages was significantly higher compared to the infection control group (*P* < 0.05). The WG in the sodium alginate encapsulation group was considerably higher than in the unencapsulated group (*P* < 0.05). Furthermore, the WG in the phage Y and G groups was significantly higher than that in the phage V group (*P* < 0.05).

**Table 3:** **Changes of weight gain of chickens in each group**

Groups	Survival rate (%)	Average weight gain (g)	Relative weight gain rate (%)
TBS	100%	95.75 ± 7.55 ^a^	100
phage Y + Alg	100%	76.82 ± 7.20 ^b^	80.23
phage G + Alg	100%	67.66 ± 7.64 ^c^	70.66
phage V + Alg	100%	50.18 ± 9.01 ^d^	52.40
phage Y	100%	60.20 ± 7.20 ^c^	62.86
phage G	100%	58.44 ± 11.88 ^c^	61.03
phage V	100%	44.63 ± 12.60 ^d^	46.61
*E. tenella*	100%	31.46 ± 4.05 ^e^	32.85
Alg	100%	32.84 ± 4.61 ^e^	34.29

Different small letters mean statistical significance (*p* < 0.05).

Weight gains were calculated as follows: BWG = body weight at 7 days post challenge − body weight when challenging.

**Figure 6:**
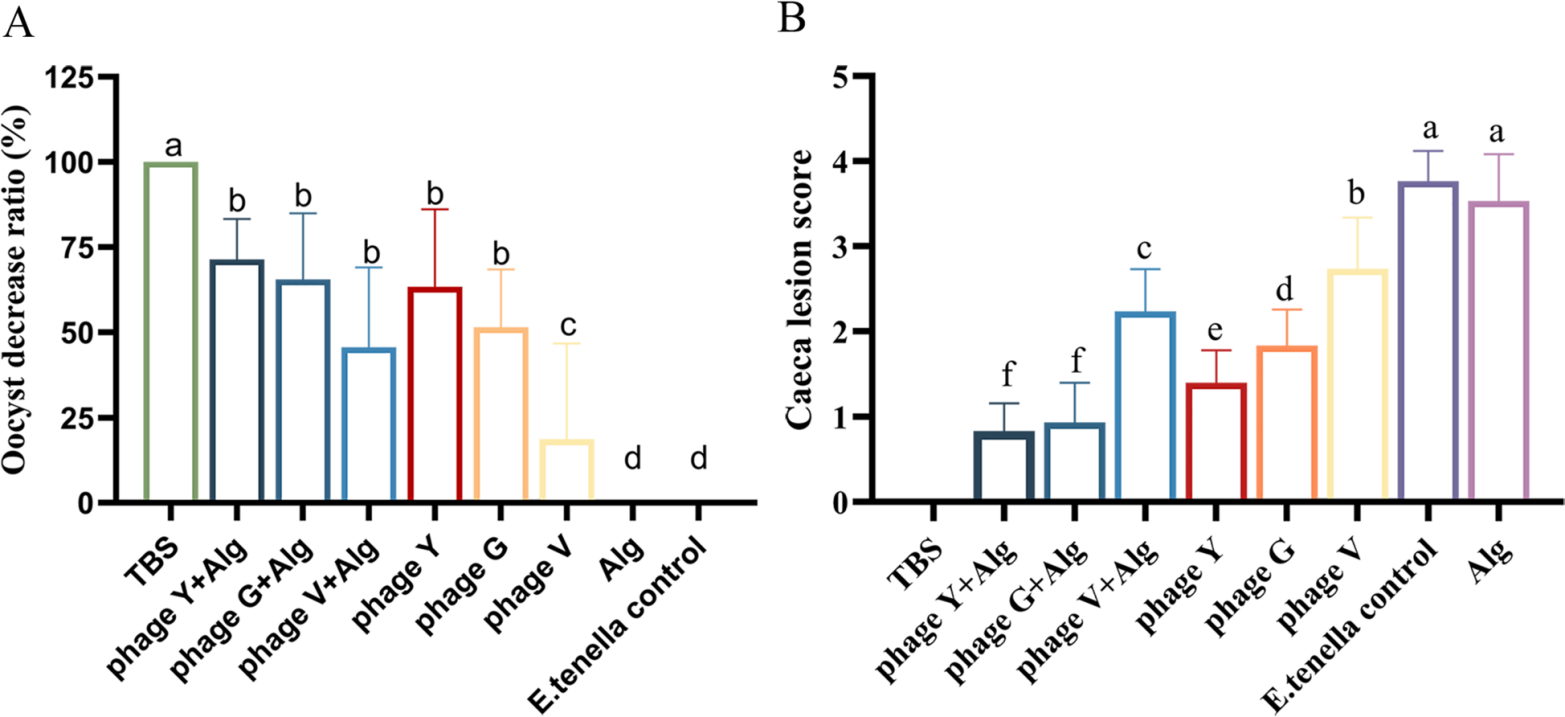
**Lesion score and oocyst decrease ratio.** 7 d after the challenge with *E. tenella,* the groups treated with phageY + Alg, phageG + Alg, phageV + Alg, phageY, and phageG showed a greater decrease in oocyst counts compared to those treated with phageV, Alg, and the infection control group (**A**). The caecal lesion scores for the phageY + Alg and phageG + Alg groups were lower than those for the phageY, phageG, phageV + Alg, and phage V groups, all of which has lower scores than the infection and Alg control groups (**B**). Values are expressed as mean ± SD (*n*** = **10). Statistical significance is indicated as follows: *P < 0.05, **P < 0.01.

The oocyst decrease ratio in the phageY + Alg, phageG + Alg, phageV + Alg, phageY and phageG groups was greater than that in the phageV, Alg, and infection control groups (Figure 6A). The cecal lesion scores in the phageY + Alg, phageG + Alg groups were lower than those in the phageY, phageG, phageV + Alg and phage V groups, which were also lower than in the infection and Alg control groups (Figure 6B).

**Figure 7:**
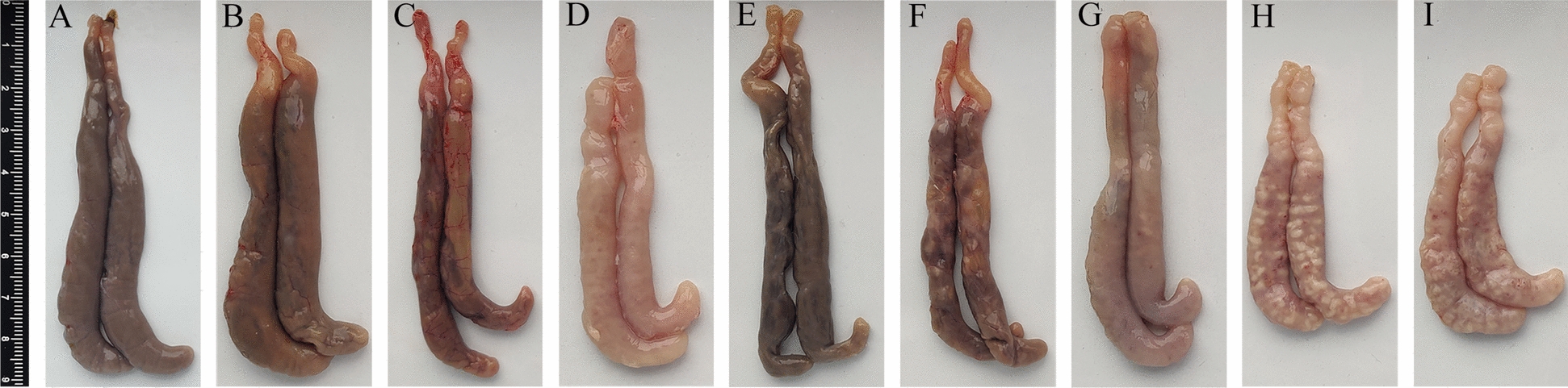
**Gross pathological lesions in ceca of chickens in each group.** 7 days after the challenge with *E. tenella*, no obvious lesions were observed in the ceca of chickens in the TBS control group (**A**). In the phageY + Alg (**B**), phageG + Alg (**C**), and phageY (**E**) groups, slight swelling of the ceca was noted. The phageG group exhibited slight caecal swelling along with a few lesions on the serosal surface (**F**). More pronounced caecal swelling with serous haemorrhagic spots was observed in both ther phageV + Alg and phageV groups (**D** and** G**). In the *E. tenella* infection group (**H**) and the Alg group (**I**), the cecal wall appeared thickened, with scattered haemorrhagic spots and grey lesions on the serosal surface.

To illustrate the protective effects, gross and histopathological changes in the ceca were documented. In the TBS group (Figure 7A), the ceca of chickens appeared normal, with no pathological changes observed. In contrast, the ceca in the *E. tenella* infection control and Alg control groups (Figures 7H and I) were small, exhibiting thickened caecal walls, scattered haemorrhage spots, and grey lesions protruding from the serous surface. Only slight caecal swelling was noted in the phageY + Alg, phageG + Alg, and phageY groups (Figures 7B, C and E). Additionally, slight cecal swelling and a few lesions on the serosal surface were observed in the phageG group (Figure 7F). In contrast, cecal swelling with serous haemorrhagic spots was observed in the phageV + Alg and phageV groups (Figures 7D and G).

**Figure 8:**
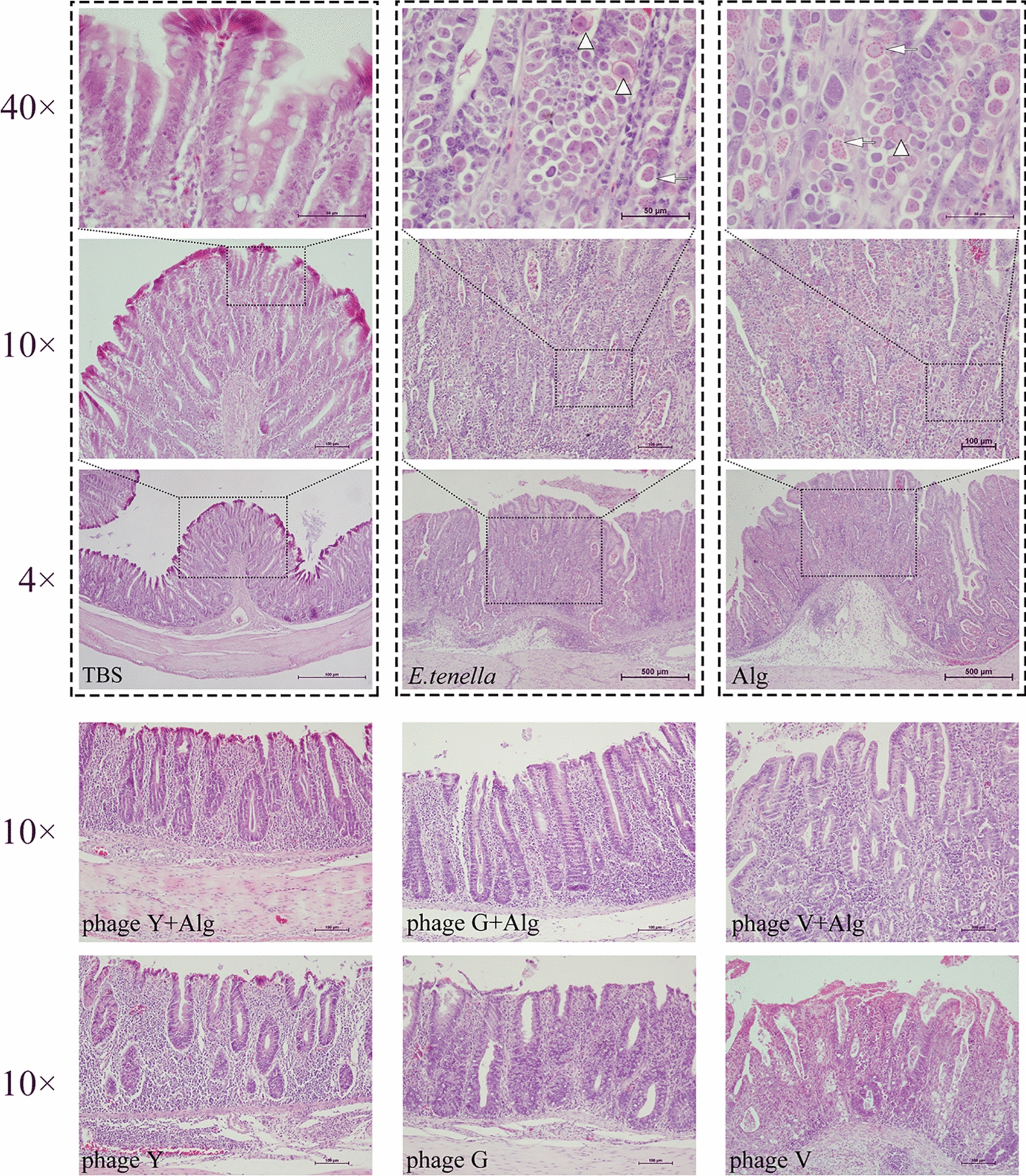
**Histopathological changes in ceca tissues from chickens in each group.** No obvious histopathological changes were observed in the ceca tissues of the TBS group. In the infection and Alg control groups, some intestinal villi were damaged, featuring infiltration of inflammatory cells in the lamina propria, oedema in the submucosa, and a marked thickening of the muscle layer. Intestinal gland epithelial cells in the lamina propria were invaded by a substantial number of *Eimeria* parasites at various developmental stages, including macrogametes, microgametes, and oocysts. In the phageY + Alg group, the structure of the intestinal villi appeared relatively normal, and the number of parasites was lower than in the *E. tenella* infection group. In the phageG + Alg, phageY, and phageG groups, the structure of the intestinal villi was partially damaged but the infiltration of inflammatory cells and the number of parasites were less compared to the *E. tenella* infection and Alg control groups. In both the phageV + Alg and phageV groups, the histopathological changes were similar to those observed in the *E. tenella* infection group.

Figure 8 demonstrates that no significant histopathological changes were observed in the cecal tissues of the TBS group. In the *E. tenella* infection and Alg control groups, partial intestinal villi were damaged, epithelial cells had shed into the enteric cavity, inflammatory cell infiltration was noted in the lamina propria, oedema in the submucosa, and the muscle layer was visibly thickened. Notably, intestinal gland epithelial cells in the lamina propria were invaded by large numbers of *E. tenella* at various developmental stages, including macrogametes, microgametes, and oocysts, which resulted in severe structural damage. In the phageY + Alg group, the structure of the intestinal villi remained normal, and the quantity of *E. tenella* parasites was significantly lower than that in the infection control group. In the phageG + Alg, phageY, and phageG groups, the structure of the intestinal villi was partially broken, with infiltrated inflammatory cells present, but the number of *E. tenella* was not as severe as in the *E. tenella* infection and Alg control group. The histopathological changes in the phageV + Alg and phageV groups were similar to those observed in the *E. tenella* infection control group (Figure 8).

### Analysis of the binding interactions between target peptides and the *Et*Cab protein through in silico methods

**Figure 9:**
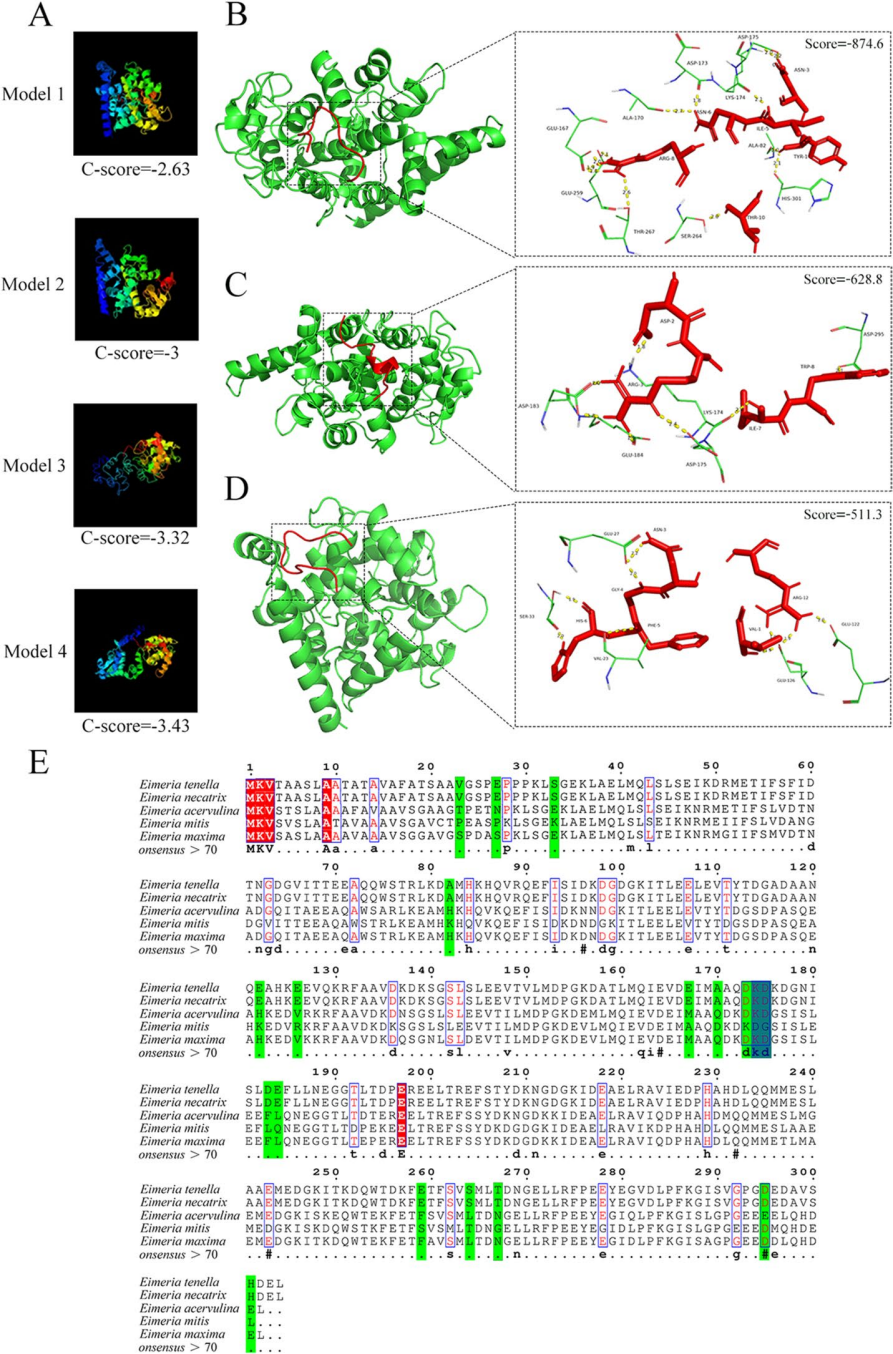
**Prediction of 3D model for EtCab protein and molecular docking with target peptides.** Molecular docking analysis was conducted to evaluate the binding of Y (**B**), G (**C**)**,** and V (**D**) peptides to the EtCab protein. The EtCab protein is shown in green, while the polypeptides are highlighted in red. Dotted lines indicate hydrogen bonds, and the numbers reflect bond lengths (with shorter bond lengths correlating to greater bond energy). The label ASN-3 specifies that position 3 contains an ASN amino acid. The I-Tasser server generated the top four models and their corresponding ratings (**A**)**.** The sequence alignment of Cab protein amino acids from five *Eimeria* species is displayed. Amino acids with a red shadow are completely conserved, those in red font red are partially conserved, amino acids bound to the short peptide are highlighted in green, and amino acids that interact with the Y and G peptides are indicated in blue (**E**).

The ProtParam web server analysis revealed that the *Et*Cab protein consists of 304 amino acid residues. A secondary structure analysis conducted using SOPMA indicated that the *Et*Cab protein contained four structural forms: 58.22% α-helix, 5.59% extension chain, 8.55% β-rotation and 27.63% random crimp (Additional file 1). The 3D structure of the *Et*Cab sequence was predicted using the I-Tasser server, along with the C-scores of the four top-ranking models (Figure 9A). The 3D model with the highest score was optimised, and its stereochemical properties were evaluated using a Ramachandran Plot (Additional file 2).

Molecular docking results demonstrated that all three affinity peptides corresponding to the three phages could bind to the *Et*Cab protein through hydrogen bonding. The binding strength was highest for the Y peptide(B) at −874.6, followed by the G peptide(C) at −628.8, and the V peptide(D) at −511.3. The amino acids involved in hydrogen bonding between the three peptides and the *Et*Cab are listed in Table 4.

**Table 4:** **Amino acids that predicted for forming hydrogen-bonds between EtCab protein and peptide Y, G and V**

Peptides corresponding to affinity phages	Number of hydrogen-bonds	Amino acids contribute for forming hydrogen-bonds in target peptides	Common amino acids in EtCab protein contribute for forming hydrogen-bonds with	
			Lys^174^ (underlined)	Asp^175^ (shadow)
Y	13	Try^1^-Ala^82^, Try^1^-His^301^, ***Asn***^3^-Asp^175^(2), Ile^5^-Lys^174^, ***Asn***^6^-Asp^173^, ***Asn***^6^-Ala^170^, ***Arg***^8^-Glu^167^(3), Arg^8^-Glu^259^, ***Arg***^8^-Thr^267^, Thr^10^-Ser^264^	●	◆
G	7	Asp^2^-Lys^174^, ***Arg***^3^-Asp^175^, ***Arg***^3^-Asp^183^(2), ***Arg***^3^-Glu^184^, Ile^7^-Lys^174^, Trp^8^-Asp^295^	●	◆
V	9	Val^1^-Glu^126^(3), ***Asn***^3^-Glu^27^, Gly^4^-Glu^27^, Phe^5^-Val^23^, His^6^-Ser^33^(2), ***Arg***^12^-Glu^122^		

Try^1^-Ala^82^ means hydrogen-bond was formed between position 1 amino acid Try in Y peptide chain and position 82 amino acid Ala in EtCab protein chain. Asn^3^-Asp^175^(2) means two hydrogen-bonds were formed between the two amino acids.

Common amino acids in three short peptides that can form hydrogen bonds with EtCab proteins are marked with italics and shadow.

To further investigate the conserved sites in the *Et*Cab protein that can be recognised by the three affinity peptides, a comparison was made of the Cab protein sequences from five *Eimeria* species: *E. tenella, E. necatrix, E. acervulina, E. mitis* and *E. maxima,* using DNAstar software. This analysis revealed that two conserved amino acids, Lys174 and Asp175, are common binding sites for both the Y and G peptides across the five *Eimeria* species (Figure 9E).

## Discussion

The invasion of *Eimeria* parasites begins with the attachment of asexual sporozoites ito host cells. Research has shown that apical membrane antigen 1 (AMA1) [32] and microneme protein 3 (MIC3) [33] both play crucial roles in this attachment process. A recent study revealed the significant involvement of calcium-binding (Cab) proteins during the invasion of apicomplexans parasites. For example, yeast expressing the *E. tenella* Cab (*Et*Cab) demonstrated a 2.5-fold higher attachment rate to host cells compared to control yeast [15], highlighting the critical role of the *Et*Cab protein in host cell attachment.

In this study, an indirect immunofluorescence assay (IFA) showed that rabbit antisera prepared against the *Et*Cab protein reacted with the proteins of sporozoites. This indicates that the antisera can bind to the natural *Et*Cab protein, consistent with previous reports [15]. Furthermore, western blot analysis confirmed that the anti-*Et*Cab antisera specifically recognised the *Et*Cab protein, as evidenced by a distinct band. However, the size of this band was larger than anticipated (about double the predicted size), suggesting the presence of post-translational modifications, such as glycosylation or phosphorylation [34], and the formation of *Et*Cab protein homodimers.

Phage display technology has been extensively used to discover peptides or antibodies specific to various pathogens or diseases, including bacteria [35–38], viruses [39–41], cancer [42, 43], and parasites [44–46]. A prior study demonstrated the specific binding of affinity peptides from *E. acervulina* and *E. tenella* to sporozoites [17]. In our laboratory, we utilised the adhesion regions (MARR) of *E. tenella* microneme protein 3 (*Et*MIC3) [7] and *Et*AMA1 proteins [8] as ligands for screening affinity peptides. The screened peptides exhibited a certain degree of anticoccidial effects.

In the present study, three identified *Et*Cab protein-affinity peptides showed strong binding capabilities and competed with rabbit anti-*Et*Cab antisera for binding to sporozoites protein and *Et*Cab protein. This suggests that the binding sites of these three target peptides overlapped with those sites recognised by the rabbit anti-*Et*Cab antisera.

Several studies have demonstrated that antibodies targeting essential proteins of *E. tenella* effectively inhibit the invasion of sporozoites into host cells in vitro [47–50]. In this study, we aimed to explore whether three identified affinity peptides could inhibit the invasion of *E. tenella* sporozoites into host cells as effectively as rabbit anti-*Et*Cab antisera. Our findings revealed that rabbit antisera to EtCab protein, diluted at a ratio of 1:100, showed similar inhibition rates to the Y and G peptides (125 μg/mL) against sporozoites invasion into MDBK cells. This observation is consistent with competitive ELISA tests, suggesting that the affinity peptides may mimic epitopes from the anti-EtCab antisera that bind to *Et*Cab proteins.

It is widely accepted that encapsulation in sodium alginate, a biocompatible and cost-effective material, can preserve phage viability after oral administration [51]. The pKa values of β-D-mannuronic acid and α-L-guluronic acid in the glycosyl chain of sodium alginate are higher than the pH value of simulated gastric juice, which facilitates the formation of hydrogen bonds. This results in a compact spherical structure that protects encapsulated phages from degradation. Conversely, in simulated intestinal juice, the pKa values are lower than thepH, causing the sodium alginate gel to swell. Phosphate and potassium ions then displace Ca^2+^ in the microspheres, gradually releasing encapsulated phages. This release process lasts approximately 1 h, aligning with the intestinal emptying time of chickens, which have short intestinal tracts.

To ensure that phages are sufficiently available at target digestive sites to efficiently bind to *E. tenella* sporozoites, we investigated the temporal and spatial distribution in the gastrointestinal tract following oral administration of encapsulated phages. The results showed that 1 h after oral administration, phage titres in the duodenum, jejunum, and ileum were higher than in the gizzard and posterior digestive tract (cecum and rectum). Within 2 h, phage titres in the cecum were slightly higher than those in the anterior duodenum and gizzard. A previous report indicated that 1 h after the infection with *E. tenella* sporulated oocysts, large numbers of free sporozoites were found in the duodenum, and 2 h later, sporozoites reached and began to invade caecal epithelial cells [2]. This suggests that the target phages have ample opportunity to contact and bind to sporozoites released in the digestive tract. We observed that orally administered phages remained detectable in the intestines 2 h post-administration, likely due to the chyme shielding the phages from digestive enzymes.

To determine whether the target phages exhibited anticoccidial effects in vivo, three different phage preparations (encapsulated and unencapsulated) were orally gavaged on days 0, 1, and 2 following oral challenge with *E. tenella* sporulated oocysts. The results indicated that the encapsulated phages were more effective than the unencapsulated ones in reducing the infection.

To clarify the mechanisms by which three target phages operate, we conducted molecular docking analysis. The analysis revealed that the Asn, Ile, and Arg residues in the three peptides associated with these three phages, particularly the Y and G peptides, can form hydrogen bonds with the *Et*Cab protein. The number and strength of these hydrogen bonds correlate with the experimental results observed in vitro and the anticoccidial effects noted in vivo.

Additionally, a sequence comparison and analysis of the Cab protein from five *Eimeria* parasites indicated that the amino acids Lys^174^ and Asp^175^ in the *Et*Cab protein interact not only with the Y peptide but also with the G peptide. This interaction explains the superior effects of the Y and G peptides observed in previous experiments.

Furthermore, we found that some of the amino acid sites in the Cab protein that bind to the three peptides are conserved across different species of *Eimeria*. This suggests that the identified affinity phages and their corresponding peptides may have broader therapeutic potential. Overall, our research provides valuable insights for developing small molecular drugs against avian coccidiosis.

## Supplementary Information

**Additional file 1**.** Prediction of secondary structure of EtCab protein.** h means alpha-helix; c means random coi; t means beta-turn; e means extended strand.

**Additional file 2**.** Optimization evaluation of EtCab model.** The red areas correspond to the “core” regions representing the most favourable combinations of phi-psi values.

## Data Availability

Not applicable.

## Abbreviations

*Et*Cab: *E. tenella* Calcium binding
NTA: nickel (Ni^2+^)-nitrilotriacetic
FITC: fluorescein isothiocyanate
SDS-PAGE: sodium dodecyl sulphate–polyacrylamide gel electrophoresis
IFA: indirect immunofluorescence assay
ELISA: enzyme-linked immunosorbent assay
HRP: horseradish peroxidase
IPTG: isopropyl-b-D-thiogalactopyranoside
SPF: specific pathogen-free
3D: three-dimensional
BWG: body weight gain
