# Supplementary figures and images for: Impact of sodium alginate hydrogel containing bacteriophage peptides that specifically bind to the EtCab protein on the inhibition of Eimeria tenella infection
Source: Vet Res. 2025 Jan 21;56:18. doi: 10.1186/s13567-024-01425-4 (PMC11752993; doi:10.1186/s13567-024-01425-4)

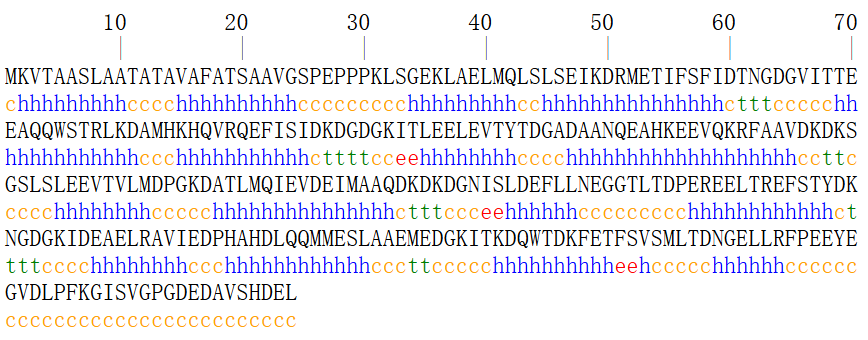

Supplement: Supplementary file 1 — Additional file 1. Prediction of secondary structure of EtCab protein. h means alpha-helix; c means random coi; t means beta-turn; e means extended strand. [file 13567_2024_1425_MOESM1_ESM.tif]

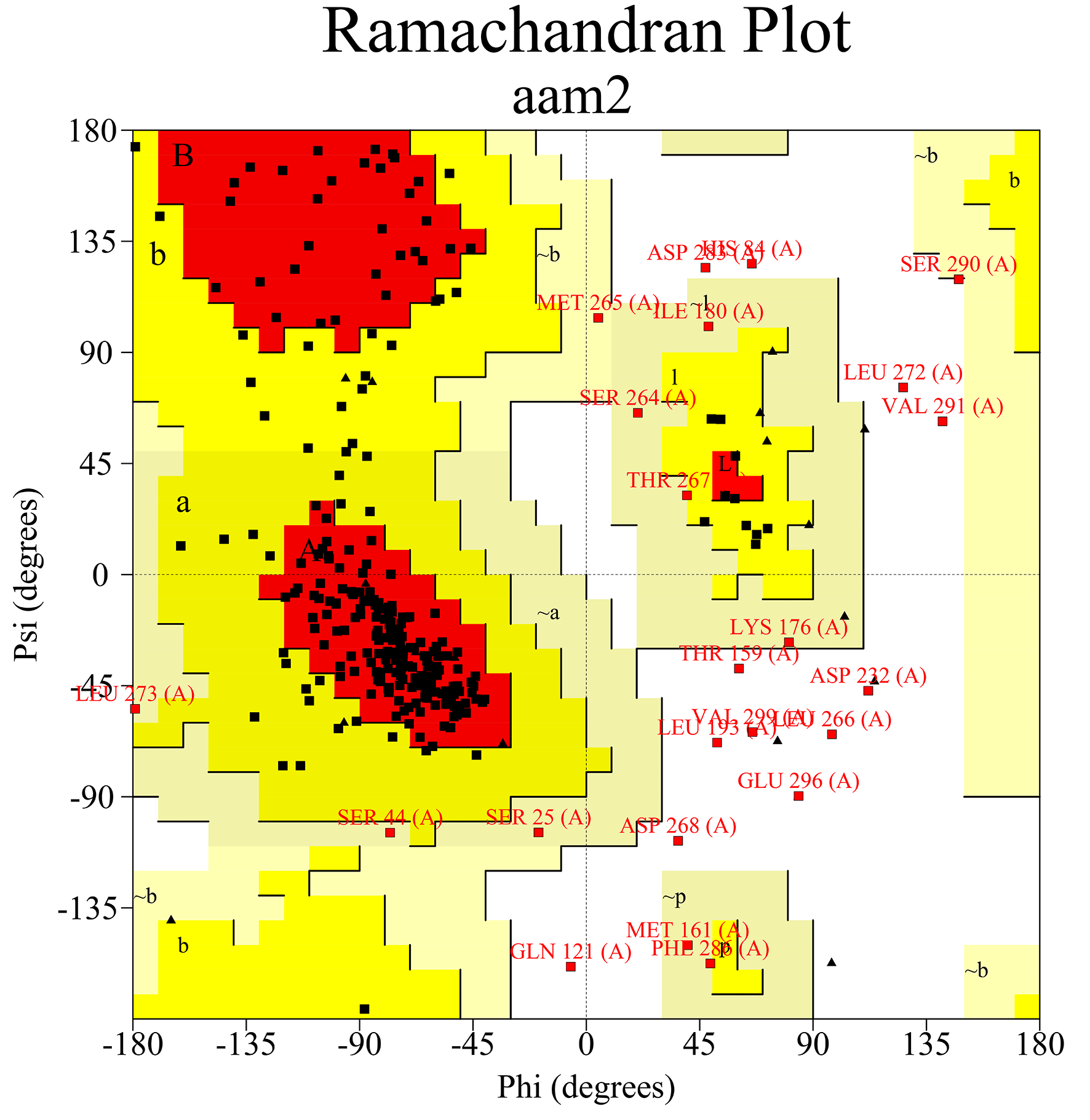

Supplement: Supplementary file 2 — Additional file 2. Optimization evaluation of EtCab model. The red areas correspond to the “core” regions representing the most favourable combinations of phi-psi values. [file 13567_2024_1425_MOESM2_ESM.tif]
